# Stakeholders’ Perspectives Regarding Supply Chain System of Pharmaceuticals and Vaccines in Pakistan: A Qualitative Study

**DOI:** 10.3390/healthcare10091738

**Published:** 2022-09-10

**Authors:** Madeeha Malik, Zeeshan Arshad, Azhar Hussain, Shazia Jamshed, Noordin Othman, Sultan Othman Alolayan, Márió Gajdács, Ibrahim Barrak, Yaser M. Alahmadi, Adeel Aslam, Sultan S. Al thagfan

**Affiliations:** 1 Department of Pharmacy Practice, Hamdard Institute of Pharmaceutical Sciences, Hamdard University, Islamabad 45550, Pakistan; 2 Department of Clinical Pharmacy and Practice, Faculty of Pharmacy, Universiti Sultan Zainal Abidin, Kuala 21300, Terengganu, Malaysia; 3 Clinical and Hospital Pharmacy Department, College of Pharmacy, Taibah University, Al-Madinah Al-Munawwarah 42353, Saudi Arabia; 4 School of Pharmacy, Management and Science University, Persiaran Olahraga Section 13, Shah Alam 40100, Selangor, Malaysia; 5 Department of Oral Biology and Experimental Dental Research, Faculty of Dentistry, University of Szeged, Tisza Lajos körút 63, 6720 Szeged, Hungary; 6 Department of Periodontology, Faculty of Dentistry, University of Szeged, Tisza Lajos körút 62-64, 6720 Szeged, Hungary; 7 Department of Pharmacy Practice, Kulliyyah of Pharmacy, International Islamic University Malaysia, Kuantan 53100, Pahang, Malaysia

**Keywords:** pharmaceuticals, vaccine, supply chain, cold chain, qualitative study, stakeholder, consumer, global health, Pakistan

## Abstract

The present study was undertaken to assess the current supply chain system of pharmaceuticals and vaccines in Pakistan in terms of structure, process, and outcomes, as well as related barriers and solutions for an effective supply chain system. A qualitative study was designed to explore stakeholders’ perceptions selected using the snowball sampling technique. A semi-structured interview guide was used to interview these respondents at a convenient time and place. After data collection, recorded interviews were transcribed verbatim and subjected to thematic analysis. The results highlighted that the standard operating procedures (SOPs), checklists, and government guidelines were available at different levels, except for community pharmacies. Timely delivery of quality products and services along with market reputation, experience, and authorization were the key criteria used for supplier selection and evaluation. Good inventory management, financial models, effective coordination, training, and skill development programs were identified as key factors responsible for an efficient supply chain process. Availability of vaccines, their appropriate temperature monitoring, and transportation are also highly compromised in Pakistan. The results of the present study concluded that the current supply chain system in Pakistan is not up to the mark; major factors include poor forecasting and inventory control, delayed order placement, lack of training, inadequate involvement of professionally qualified staff, inadequate financing and procurement processes, and poor coordination and integration among all stakeholders.

## 1. Introduction

Demand patterns are fluctuating with increased life expectancy, literacy rates, and incomes, as well as better awareness of health-related issues, creating greater demand for pharmaceutical products in developing countries [1]. Similarly, supply-side dynamics are also changing. Keeping these in mind, developed countries have shifted focus to biologics, which has generated opportunities for developing countries to fill the gap for production of cost-efficient, competing small-molecule therapeutics, especially for high-quality me-too generics, super-generics, and simpler biologics such as vaccines and antisera [2]. Due to decreasing product life cycle time, varying customer demands, and the increasing cost of manufacturing and shipment, manufacturers have focused more on efficient supply chain management to reach organizational goals via speeding up innovations and product launching to the dynamic market, improving customer value, optimizing utilization of resources, decreasing different types of costs such as production, inventory, and transportation, and increasing profitability. Supply chain efficiency is important in terms of cost and asset management. As with efficient supply chain management, the costs of production, inventory, and transportation are reduced, and customer service levels are improved. As companies augment the costs, they achieve higher efficiency and create more efficient relationships with partners by increasing forecast accuracy and repeatability, as well as by adopting just-in-time delivery strategies for their high-cost raw materials. These activities result in a significant decrease in indirect costs [3]. A study by Oliver Eitelwein highlighted that many pharmaceutical companies have to increase the main supply chain sections including customer satisfaction, forecasting accuracy, inventory level, and total supply chain costs [4]. Furthermore, a large finished good portfolio, the wide variety of materials needed, distribution networks, high investment cost and time of developing new products, capacity constraints, and regulatory restrictions were also indicated as important factors influencing supply chain management [5].

The concept of pharmaceutical supply chain management envisages the distribution of good-quality pharmaceutical products among the consumers at the right place and time. The traditional supply chain management model is mainly associated with the application and most effective use of resources. Advancements in pharmaceutical supply chain processes have changed the concept of healthcare systems and health management over the last decade [6]. Planning material requirements, flexible manufacturing systems, quality management, and just-in-time methods have focused on effective supply chain systems. Furthermore, key factors in supply chain management include inventory management that requires a balance between services needed by consumers; availability of products, and cost-effectiveness [7]. Multiple factors and risks may lead to the vulnerability of pharmaceutical supply chains, including resource wastage and drug stockouts [8]. However, the primary causes responsible for drug shortages are complex and may include different issues related to manufacturing, regulatory perspectives and policies, supply and demand, distribution, natural disasters, inventory, and workforce at different levels of the supply chain [9]. Allocation of limited budgets to sustain healthcare systems in developing countries is considered a significant challenge faced in providing access to medicines. Poor pharmaceutical supply chain management is regarded as one of the primary obstacle in providing adequate healthcare facilities. In addition, such healthcare systems face major issues of drug and vaccines stockouts due to inefficient pharmaceutical supplies [10].

Shaojian Qu et al. proposed an improved genetic algorithm of mixed-integer robust maximum expert consensus models (MIR-MECMs) in large-scale group decision making (GDM), and a simulation analysis showed that the ordered weighed averaging (OWA) operator had a more stable performance with parameter perturbation [11]. Similarly, Ying Ji et al. determined the way to reach a consensus under a risky and uncertain environment by taking into account the existing knowledge of GDM through a combination with the related development of two-stage stochastic programming and risk measurement, as well as the proposed modeling method of two-stage mean-risk stochastic minimum cost consensus. Moreover, a study reported that, from 154 vaccine supply assessments in 89 countries conducted between 2009 and 2016, the performance of nine indicators mostly fell below the recommended 80% threshold [12].

Pakistan possesses a local market of 215 million consumers, and more than 700 pharmaceutical companies are positioned well to gain from opportunities provided under these shuffling global patterns of supply and demand. However, the existing practice of simply importing 95% of the raw material, compounding active ingredients with excipients, coating the pills, and packaging the drugs cannot continue to be the long-term goal of the sector. Industry stakeholders feel that Pakistan’s current exports of 218 million USD can easily cross 0.5 billion USD in the coming years with the focus on introducing effective and timely measures to overcome gaps in the supply chain that could help them gain substantial international market share [13]. Certain features have hindered the progression of the pharmaceutical market to an advanced sector including increased market share of large companies, high prices controlled with excessive Drug Regulatory Authority of Pakistan (DRAP) regulations, weak regulations and enforcement of standards, and poor supply chain mechanism with reduced efficiency, skills and technology transfer, investment, quality, and capacity upgradation. Major challenges identified in distributing and accessing essential medicines during the humanitarian crisis following the 2005 earthquake, floods (2010), and internally displaced people (2011) were poor-quality pharmaceuticals and medicine supply shortages. Two major incidences of poor-quality medicines in Pakistan claimed the lives of hundreds of people in 2011 and 2012. These incidences include the case of contaminated cardiovascular drugs in December 2011 which claimed more than 230 lives (“the fake drug crisis”), while another major case of medicine quality failure occurred, causing the death of hundreds of people after ingesting contaminated cough syrup. A drug distribution network with early detection and rectification of quality and supply failures in the affected areas was established by the World Health Organization (WHO) in 2005 in Pakistan. The WHO prequalification program was adopted countrywide by a number of organizations leading to improved delivery of quality medicines. However, a study from Pakistan reported no significant benefit in terms of time taken for smear conversion for the 15–20% more expensive, internationally quality assured medicines when compared with locally produced multidrug-resistant tuberculosis medicines purchased through the medicine prequalification program. In this way, new control strategies have been developed, including the interventions at a system level rather than being limited to punishments and penalties [14].

Effective and efficient pharmaceutical supply chain management has always remained a major challenge that Pakistan needs to address. The foremost factor responsible for this inconsistent pharmaceutical supply chain is the lack of synchronization among those performing their duties in the drug supply chain and those involved in inventory management [15]. Moreover, lack of coordination of demand information at various stages of the chain, dependency on human resources in the field of the supply chain of medicines, legislation issues related to manufacturing, distribution problems, demand and supply discrepancies, management of orders, drug shortages, expiry, and warehouse management also contribute toward an inconsistent supply chain [16,17,18]. A well-structured and integrated action plan for procurement, production, and distribution activities for achieving competitive advantage in today’s marketplace is required in Pakistan. Supply chain challenges in the health sector are complex and provide specific and highly visible opportunities for quick returns on investment (ROIs). Supply chain reform may act as a catalyst for massive health system reform [19]. However, supply chain reform requires strong and objective evidence coupled with serious feasibility and implement ability analysis, which is not the case for Pakistan. Although there have been few well-designed randomized or quasi-randomized studies highlighting pathways to improve supply chain performance within health systems in the developed world, more data need to be generated in the case of developing countries, including Pakistan. Due to the context-specific nature of supply chains and their dependence of multiple external factors, it is difficult to rely on studies from other contexts because their external validity is quite limited in the local context of any country. Limited studies have been conducted to produce evidence-based data to understand better the supply chain system of pharmaceutical products and vaccines in this country. This can help to produce basic supply chain theory and modeling for Pakistan, helping to generate the right supply chain data, which can be properly analyzed to provide the information needed to make supply chain improvement decisions. Therefore, the present study was designed to assess the current supply chain system of pharmaceuticals and vaccines in Pakistan in terms of structure, process, and outcomes, as well as related barriers and solutions for the effective supply chain system from the perspectives of stakeholders in this area.

## 2. Materials and Methods

### 2.1. Study Design

A qualitative study was designed to explore the perceptions of different stakeholders including regulators, manufacturers, distributors, and hospital and community pharmacists regarding factors affecting supply chain management of pharmaceutical products and vaccines in Pakistan.

### 2.2. Study Site and Respondents

For this purpose, semi-structured interviews were conducted with chief executives, supply chain managers, procurement officers, and professionally qualified pharmacists working in manufacturing companies, distributors, retailers, and hospitals. The interviewees were selected using the purposive and snowball sampling method. First, we interviewed the officials at the Drug Regulatory Authority of Pakistan (DRAP); then, we asked them to introduce people of interest in this field. We then selected the study participants from well-informed individuals who were willing to share their opinions. The inclusion criteria were: sufficient information, knowledge, and experience about the storage, distribution, and delivery of medicines and the challenges of the pharmaceutical supply chain. The study sites for this research included pharmaceutical manufacturing industries, distributors, public and private hospitals, and community pharmacies located in the twin cities of Islamabad (population ~1.05 million) and Rawalpindi (population ~2.10 million) in Pakistan [20].

### 2.3. Sample Size, Sampling Technique, and Data Collection

For data collection, individual interviews were conducted with participants, preferably at their workplaces by arrangement. The interviews were conducted in Urdu, as this was the formal language and mother tongue of all participants. At the beginning of the interviews, general explanations about the aims of the study and the need for confidentiality of information were given verbally. Written informed consent was also obtained from all interviewees, and it was ensured that they could withdraw from the study at any time. Interviews lasted a minimum of 30 min. All interviews were conducted by one of the researchers. They were recorded with the consent of the participants and transcribed within a very short time after completion. Interviews continued until saturation, which was reached for different respondents after interviews such as regulators (*n* = 6), manufacturers (*n* = 9), distributors (*n* = 13), hospital pharmacists (*n* = 13), and community pharmacists (*n* = 15). The saturation level in qualitative studies is when no new concept can emerge, and the latest data added to the previous data are simply a duplication. As data collection and data analysis were simultaneous, the saturation level of the available data was reached, and the process of data collection was interrupted. A snowball sampling technique was adopted for this study as it is the best way of identifying the respondents having common characteristics, experience, and job profiles [21]. For scientific validity, the researchers took into account procedural rigor and sample representativeness followed by transferability and generalizability.

### 2.4. Data Collection Tool, Reliability, and Validity

In preparing the semi-structured interview guide, a review of the relevant literature was conducted along with an open-ended pilot interview; it was then confirmed by two experts of supply chain management from academia. In addition, the validity of the interview guide was confirmed after conducting two pilot interviews with participants. To increase the accuracy and precision of the study, four criteria developed by Guba and Lincoln were considered, namely, credibility, conformability, transferability, and reliability [22]. A demographic questionnaire was administered to the respondents before conduction of the interview. The interview guide used comprised three major sections: (a) the first section was regarding supply chain processes, consisting of 12 questions; (b) the second section was regarding supply chain indicators, comprising nine questions; (c) the third section was about outcomes and consisted of five questions. To increase the credibility of the study, long-term participation and continuous observation were used so that the researchers were fully involved in the study, appropriate communication was established with the participants, and general concepts that emerged during the study were accepted. In addition, interviews and reviews of the related literature were integrated to accomplish this purpose. To increase the conformability of the findings, coded data were presented to the participants to verify their accuracy. The transferability of the study results was confirmed by explaining the conditions of the informed study participants and the interview method in an understandable manner. An attempt was also made to fully select the participants according to the study’s objectives and our primary inclusion criteria. The data were analyzed simultaneously in parallel with data collection to help the researchers be fully informed about the principles of theoretical research. To increase the reliability of the study findings, the concepts, themes, and audio and textual information were coded. To ensure this, two members of the research team analyzed the content individually. They discussed the themes to reach a consensus in case of disagreement. When the coding process was complete, all initial codes were discussed by all research team members who had no conflict of interest regarding the topic. Lastly, two qualitative experts finalized the codes.

### 2.5. Data Analysis

Data analysis was conducted in five steps. First, the researcher listened to the audio files of the interview sessions, and the transcribed texts were read to identify the data. In the second step and to identify a thematic framework, repetitive ideas were reduced into groups of similar ideas or codes in the process of identification. In other words, through a decreasing process, the researchers grouped the whole text into meaningful units that reflected the answer to the research question. Then, the initial codes were extracted from those meaningful units. Third, indexing units or portions of the data associated with a particular code were characterized. As mentioned earlier, the initial codes were then discussed by all members of the research team and two qualitative experts to arrive at the final codes. In the fourth step, the data were summarized in a table according to the thematic framework, which included the final codes, subthemes, and main themes. Lastly, in the fifth step, the data were combined. Coding and classification of data were performed using the InVivo software (DEXIS, Hatfield, PA, USA). All transcribed interview content was translated into English for better use of the data analysis software [23]. Bastani et al. and Bastani et al. adopted a similar methodology for their qualitative study on the assessment of supply chain management of pharmaceuticals in Iran [24,25].

### 2.6. Ethical Considerations

Ethical approval was sought from the ethical committee of Hamdard University prior to the commencement of this research (approval number: BASAR-119-ERB-HUIC-039-Jan-2018). Written informed consent was obtained from the respondents before conducting the interview. They were briefed about the research objectives, privacy, and confidentiality of their data. The respondents were made aware that their participation in the research was voluntary, and that they could withdraw from the study at any time.

## 3. Results

### 3.1. Demographic Characteristics

Among the total respondents working as regulators, 16.7% (*n* = 1) were working at the National Institute of Health, 33.3% (*n* = 2) were working in the drug regulatory authority of Pakistan, and 50% (*n* = 3) were working at the district health office. Among distributors, 7.7% (*n* = 1) were working as supply chain managers, 15.4% (*n* = 2) were working as marketing managers, 15.4% (*n* = 2) were working as general managers, 15.4% (*n* = 2) were working as managing directors, 23.1% (*n* = 3) were working as institutional managers, and 23.1% (*n* = 3) were working as chief executive officers. Among hospital pharmacists, 15.4% (*n* = 2) were working as senior hospital pharmacists, 23.1% (*n* = 3) were working as procurement officers, and 61.54% (*n* = 8) were working as hospital pharmacists. A detailed description of the respondents’ composition is given in Supplementary Table S1 (Regt: regulator, Mfr: manufacturers, Dist: distributor, HP: hospital pharmacist, CP: community pharmacist).

### 3.2. Theme 1: Operational Framework for Supply Chain Management

#### 3.2.1. Availability and Updating of Government Policies/Guidelines

Most of the regulators and manufacturers stated that the government has devised different policies and regulations for the procurement of pharmaceutical products and vaccines in Pakistan (Table 1). On the other hand, all the distributors highlighted a lack of national policies and procedures related to the procurement of pharmaceuticals products and vaccines at their level. Most of the hospital pharmacists indicated that there were policies available at the government level, while few of them believed that generalized rules are available; some of them stated that the government gives no such policy, and that they were following the policies developed on their own. Moreover, all the community pharmacists believed that no government guideline or policy for the procurement of pharmaceutical products and vaccines at the level of community pharmacy was available.

“*Different guidelines for procurement of pharmaceuticals and vaccines are devised by the government, in which tendering process for bulk procurement with each and every step regarding procurement is well explained.*”

(Regt 06)

“*Yes, we do have rules and guidelines to follow. The Drug Regulatory Authority of Pakistan (DRAP) has devised import and export rules, the requirement of form 5, form 7, certificate of analysis, airway bill, and affidavit.*”

(Mfr 09)

“*We follow the DRAP guidelines in terms of having a drug distribution license. Besides this, I don’t think there are any government policies or regulations available for pharmaceutical procurement.*”

(Dist 01)

“*Yes, the government has provided us detailed Punjab policies, i.e., the LP policy of Punjab including tendering process, supplier selection criteria, and procurement process.*”

(HP 06)

“*Although we have Public Procurement Regulatory Authority (PPRA) rules, these are generalized procurement rules, and except these, we don’t have any rules and policies for vaccines and pharmaceuticals procurement. Especially for vaccine procurement and storage, no specific rules and policy exist as per my knowledge.*”

(HP 11)

“*As per my knowledge, we don’t have any policy or guideline from the government regarding procurement at the pharmacy level, and we never get any standard operating procedures (SOPs) from DRAP or any other regulatory body regarding procurement.*”

(CP 02)

Data of important supply chain indicators affecting supply chain management retrieved by using the interview guide were compiled in perspective of all the stakeholders for comparison (Table 1).

#### 3.2.2. Availability of Standard Operating Procedures (SOPs)

Almost all of the regulators stated that they had generalized rules and regulations, and that they were following them in their routine practice. However, some of them also highlighted that, although generalized SOPs were present, such procedures specifically related to the supply chain were missing. All manufacturers agreed that SOPs were available for every department in the pharmaceutical manufacturing industry. Almost all the CEOs and managers of distributors stated that, for ordering, stock handling, delivering, receiving, and temperature monitoring, they designed SOPs that were well communicated to their employees. A few highlighted that they were also following the World Health Organization (WHO) guidelines. All the hospital pharmacists agreed that they had different SOPs available according to their nature of work, which they implemented and followed in their routine practices. On the other hand, most of the community pharmacists highlighted that they did not have any SOPs available at their pharmacies; a few indicated that they had SOPs related to inventory management, order placement, receiving, and delivery of supplies.

“*We do have general rules and regulations, and we are following these, but in addition to that, in DRAP, every department has made its own internal SOPs depending on their workload and are following them to do the work in a systematic way.*”

(Regt 04)

“*We are following both national and international SOPs; we have very well-defined standard operating procedures, and, in many cases, we also follow WHO guidelines.*”

(Regt 06)

“*Yes, we do have well-documented SOPs and instructions. All of us follow them and never compromise over them.*”

(Mfr 06)

“*We have well-defined policies and SOPs. However, we also follow WHO guidelines for procurement customized according to our own requirements.*”

(Dist 03)

“*We do have written SOPs for demand and supply and follow them accordingly.*”

(HP 05)

“*We don’t have any SOPs. We manually check our shelves for stock level, note it down, and then place the order.*”

(CP 03)

#### 3.2.3. Availability of Checklists

Most of the regulators and manufacturers stated that a checklist was used to ensure compliance of supply chain processes with standard guidelines. Quality control departments and compliance managers were responsible for assuring that the whole process is thoroughly followed and implemented. Multiple tools were being utilized at distribution, hospital, and community setups to ensure that each and every step involved in the supply chain process was followed entirely.

“*We are using a checklist to ensure the compliance of the supply chain process.*”

(Regt 05)

“*In the manufacturing industry, all steps and units are interlinked, and it is challenging to avoid any single step. We ensure that all of our customers get their demanded stock in time. We ensure availability of raw materials in our warehouse for timely delivery.*”

(Mfr 03)

“*We have multiple steps for the verification of stock and temperature monitoring. In and out forms are used to ensure compliance with standards. The customer is informed after dispatch of products, and follow-up is also carried out until receiving of products.*”

(Dist 03)

“*We are using an HMIS (hospital management information system) in our hospital, so every step is verified in terms of completion before moving on to the next step.*”

(HP 01)

“*We are highly equipped in technology. We have multipurpose software for alerts at minimum levels, ordering, checks on expiries, and controlled drug dispensing. Besides this, our manager of Outpatient Departments (OPDS) also ensures that every step is completely followed.*”

(CP 01)

### 3.3. Theme 2: Key Indicators for Supply Chain Management

#### 3.3.1. Selection Criteria for Local and Foreign Suppliers

The regulators agreed that the selection criteria were available for suppliers at a national level. The manufacturers highlighted that certification from relevant bodies, the quality of services, market reputation, and timely delivery of raw materials were the main selection criteria for them. A valid license to sell pharmaceuticals, distribution profile, experience certificates from those institutions where they are already doing business as suppliers, bank statements, authority letters from parent companies, and credit-bearing capacity were the defined criteria for the selection of suppliers at a distribution level. Hospital pharmacists defined previous history, experience, financial conditions, authorization, and registration with a licensing authority as criteria for supplier selection. Most of the community pharmacists highlighted that they did not have any criteria to select a supplier. The pharmaceutical companies decide who they authorize for their products. At the same time, some pharmacists stated that they chose a supplier on the basis of the number of pharmaceuticals they were dealing.

“*The vendor prequalification and selection criteria across the country include vendor technical expertise, their resources, market repute, and work commitment for delivering the order in time.*”

(Regt 01)

“*For supplier selection, the foremost thing is that they must have a Food and Drug Administration (FDA) approval or if not—as in the case of China—then they must have valid Good Manufacturing Practice (GMP) certification. Besides this, we also check that they provide quality services, and raw material should meet our standards.*”

(Mfr 05)

“*Institutions ask for information, including valid drug distribution license, experience certificates, and financial statements on an affidavit.*”

(Dist 06)

“*While selecting a supplier, we do ask about their experience, financial stability, and performance certificate from the institute where they are already supplying their products, an authority letter from the parent company, and a valid drug sale license.*”

(HP 01)

“*There is as such no criteria for community pharmacies. However, it depends upon the prescription of any product and on an authorized distributor for supply by the parent company.*”

(CP 09)

#### 3.3.2. Availability of Performance Evaluation Criteria for Supplier

Almost all the regulators and manufacturers explained delivery of quality products, timely provision of ordered stock, professionalism, ethics, and market reputation of suppliers as the key criteria for the performance evaluation of suppliers. Almost all the distribution representatives, as well as hospital and community pharmacists, believed that supplier performance is evaluated against their timely delivery of supplies, facilitation in breakages, expiry management, and their support/flexibility in delayed payments by the institute.

“*Performance of suppliers is evaluated on the basis of their timely delivery of supplies, quality of services provided by them, and their professional attitude with the organization.*”

(Regt 03)

“*It is evaluated on the basis of the services, quality, and on-time delivery of the ordered consignments.*”

(Mfr 01)

“*The institute is mostly evaluating the performance of supplier on the basis of their timely services for demanded products, professionalism, and proper documentation.*”

(Dist 03)

“*Performance is evaluated on the basis of on-time delivering of the supplies.*”

(HP 02)

“*Their performance is evaluated by their timely visits, deliveries, and management of expiry issues.*”

(CP 04)

### 3.4. Theme 3: Factors Affecting Supply Chain Management in Pakistan

#### 3.4.1. The Procurement Process

The procurement process is a critical factor that affects supply chain management in Pakistan. Moreover, accurate lead time calculation and efficient forecasting play significant roles in the supply chain process, which all regulators highlighted. Almost all manufacturing pharmacists stated that efficient supply chain management is the outcome of good inventory management and procurement process. All of the distribution, hospital, and community respondents believed that efficient procurement, inventory management, and forecasting result in an efficient supply chain process.

“*Inappropriate forecasting leads toward excess or understocking of products. Similarly, inaccurate lead time calculation results in the delayed supply of products leading to stocks being out, affecting supply chain management.*”

(Regt 01)

“*We try to maintain quarterly or sometimes 6 months worth of stocks of raw material. Procurement plays a major role as if a team is not vigilant and if a poor forecasting and quantification method is used, then it will affect supply chain process.*”

(Mfr 01)

“*Inventory management and forecasting directly affect the consistency of supply. If procurement or forecasting methods are inappropriate, you can’t achieve consistency in supplies.*”

(Dist 03)

“*In this regard, our pharmacy store department is crucial. If they do not maintain the inventory level according to 3 months consumption, it can definitely affect the procurement and supply process.*”

(HP 01)

“*Procurement processes and their management do affect the consistency of supplies.*”

(CP 07)

#### 3.4.2. Available Financial Resources

Almost all the regulators, manufacturers, distributors, hospitals, and community pharmacists believed that the financial strength of a firm is directly proportional to the efficiency of its supply chain management system.

“*Finance is the backbone of every process and is directly proportional to an effective supply chain management processes. Limited or a lack of finances can disrupt the supply chain management.*”

(Regt 03)

“*You will always be on a supplier’s priority list if your payment mode is either in the advance form or an efficient payback system, which will directly have a positive impact on the supply chain process.*”

(Mfr 03)

“*I must say finance is the key factor for a sound supply chain. I will give you an example of the Capital Development Authority (CDA) hospital, and there are many financial issues, so the supplier—irrespective of the fact that they get selected after the tender process—they don’t supply products to the CDA after their credit amount reaches a certain limit.*”

(Dist 01)

“*Availability of finance directly influences the timely availability of supplies. I would like to highlight that, when an institute does not have any financial issues and the supplier is getting their payments without any delay, they will keep that institute on their priority list for delivery of products.*”

(HP 13)

“*Finances are directly proportional to the timely availability of supplies.*”

(Pharm 01)

“*If the pharmacy is financially sound then it will get on-time supplies, as the suppliers avoid those pharmacies where they face payment issues.*”

(CP 06)

#### 3.4.3. Supplier–Institute Relationship

All the stakeholders highlighted that a good collaboration and coordination among suppliers and institutions are required to strengthen supply chain management processes in Pakistan.

“*The supplier-and-institute relationship affects the supply chain process. Suppose the institute does not have a good relationship with the supplier. In that case, it will result in a lack of effective coordination, leading to miscalculation of lead and product quantities, which adversely affects the supply chain management process.*”

(Regt 01)

“*In our field, a positive relationship between supplier and institute counts a lot; one cannot do business without this.*”

(Mfr 02)

“*In our field, it counts a lot, as one cannot do business without a positive relationship.*”

(Dist 04).

“*A positive relationship between buyer and supplier will result in a positive influence on supply chain services.*”

(HP 01)

“*The supplier–institute relationship is directly proportional to the efficient supply chain. A good buyer-and-supplier relationship results in a consistent supply chain process.*”

(CP 02)

#### 3.4.4. Consumer Satisfaction

Almost all of the stakeholders were of the view that consumer satisfaction is the outcome of a good supply chain process and directly affects the supply chain management system.

“*Definitely, consumer satisfaction affects the outcomes of the supply chain process.*”

(Regt 02)

“*If your product is not of good quality and does not fulfill its purpose, then it will definitely affect consumer satisfaction.*”

(Mfr 03)

“*Consumer satisfaction directly affects the outcomes of the supply chain process. If the consumer is not satisfied, then they will get the alternative from other sources.*”

(Dist 03)

“*I must say that the outcome of an effective supply chain process depends on consumer satisfaction.*”

(HP 02)

“*Yes, consumer satisfaction affects the outcome, especially when we talk about pharmacies as a customer has multiple available pharmacies.*”

(CP 05)

#### 3.4.5. Training and Skill Development Programs

Most of the regulators highlighted that training and skill development programs on supply chain management are not available in public sector organizations. However, a few of them indicated that they had attended different trainings and skill development programs on supply chain management on their own accord. Most of the stakeholders highlighted a lack of training and skill development programs in supply chain management at their levels, except for manufacturers.

**“***In the majority of the public sector organization, training and skill development programs for supply chain management are not available.*”

(Regt 01)

“*We do training and skill development programs of our whole staff on a regular basis as it is a requirement of GMP.*”

(Mfr 04)

“*We don’t have proper training at the distribution level, but we do guide our employ for stock handling, delivery, and temperature monitoring and, in my opinion, these are the only requirement at distribution level.*”

(Dist 01)

“*We do have regular training conducted at all levels.*”

(Hosp 06)

“*Training and skill development programs for supply chain management must be included but, unfortunately, we don’t have them in routine practice due to which we face serious issues related to supply chain management which could be addressed through proper training.*”

(HP 02)

“*No, we don’t have any mechanism of training at pharmacies. We learn the skills of purchasing with experience.*”

(CP 12)

### 3.5. Theme 4: Outcomes of Efficient Supply Chain Management

#### 3.5.1. Improved Healthcare Outcomes

Almost all of the stakeholders described improved health outcomes which resulted from efficient supply chain management.

“*When the therapy is provided within the prescribed time, costs will be reduced, resulting in improved healthcare outcomes.*”

(Regt 04)

“*Definitely, efficient pharmaceutical supply chain improves health outcomes, as, when standards are followed and quality of the material is maintained, it eventually results in improved health outcomes.*”

(Mfr 07)

“*An efficient supply chain system improves health outcomes. If medicines are available on time, resources are utilized in a better way.*”

(Dist 04)

“*Definitely, an effective and efficient pharmaceutical supply chain can improve health outcomes. When quality and timely availability of supplies are ensured through an efficient pharmaceutical supply chain, it will directly improve health outcomes.*”

(HP 04)

“*In my opinion effective and efficient pharmaceutical supply chain can improve the health outcomes.*”

(Pharm 05)

#### 3.5.2. Reduced Healthcare Costs

All of the stakeholders were of the view that healthcare costs might be reduced with the help of an effective supply chain management process.

**“***An efficient supply chain management results in cost reduction. For instance, if your vendor selection is correct, then you can procure a cost-effective product in time. Similarly, appropriate forecasting will reduce the problem of stocks being out and save on emergency procurement, which is more expensive. Thus, well-established supply chain management systems, including appropriate forecasting, inventory, and order, can save on the costs.*”

(Regt 01)

“*An efficient pharmaceutical supply chain results in cost reduction. If raw materials are available on time at reasonable rates, then cost will be saved and overall healthcare cost will be reduced.*”

(Mfr 05)

“*It plays a major role in cost saving as if supplies are delayed, and then late delivery charges are applied which cut down the profit margin.*”

(Dist 05)

“*Pharmaceutical products are brought through a tender at a reasonable price. If the supply chain is not efficient, then drug shortages are faced, and products are purchased at increased prices in emergency situations.*”

(HP 01)

“*Yes, an efficient pharmaceutical supply chain management will always play a major role in cost saving.*”

(CP 04)

### 3.6. Theme 5: Outcomes of Efficient Supply Chain Management

#### 3.6.1. Issues Related to Import and Availability of Vaccines

Almost all of the regulators were of the opinion that there are various issues around the import and availability of vaccines. Poor pricing policies for vaccine import and DRAP limitations for import of vaccines from India were the key issues highlighted by most of the manufacturers. Most of the distribution and hospital respondents stated that there were many issues regarding the import and availability of vaccines, including poor pricing policies, inventory management, and complex import processes. On the other hand, community pharmacists believed that vaccines are products relevant for hospitals (they are administered there), and there is limited demand for vaccines at community pharmacies. However, when an order is placed for vaccines, most of the community pharmacists more commonly communicated unavailability.

“*The availability of vaccines is a hot issue in Pakistan; we are the only manufacturer of vaccines at the government level, and it’s really difficult to meet the requirement of vaccines at the national level. Factors associated with vaccine unavailability are raw material availability issues, inappropriate quantification methods, dollar price fluctuation, and a lengthy selection procedure for alternative foreign vaccine/raw material supplier selection.*”

(Regt 06)

“*In Pakistan, very limited vaccines are manufactured; most of the vaccines are imported from different countries. Due to the bank, customs, and DRAP’s complex policies and regulatory requirements regarding import of vaccines, we face availability issues nationwide.*”

(Mfr 01)

“*Shortage of vaccines is one of the biggest issues in Pakistan and is one of the prime concerns at present.*”

(Dist 09)

“*There are a number of requirements of DRAP and the customs department for the import of vaccines, which take a lot of time to release, and at the time we also face vaccine stock-outs which is a big challenge in Pakistan.*”

(HP 01)

“*Vaccines are mostly demanded, procured, and dispensed at a hospital level, and we have very limited demand of vaccines at community pharmacies. Whenever we demand vaccines for sale, their availability always remains an issue.*”

(CP 03)

#### 3.6.2. Storage and Temperature Control of Vaccines

There are gaps in pharmaceutical rules and regulations regarding vaccine storage and temperature control during transportation. Vaccines—being highly sensitive to temperature exposure—face challenges of proper storage and temperature monitoring facilities, which most of the regulators highlighted. Most of the respondents working in the manufacturing sector believed that most vaccines are imported from different countries, and we do not have a proper legal requirement to monitor the temperature of vaccines during the transportation and storage phase. Due to inappropriate policies regarding distribution requirements of vaccines, as well as temperature monitoring of vaccines during storage and transportation, vaccine quality can be compromised; this was highlighted by most distributors, hospitals, and community pharmacists.

**“***Storage and temperature monitoring of vaccines is conducted under the supervision of a qualified pharmacist. Different methods have been devised for temperature monitoring during transportation. One of them is the VVM method (vaccine vial monitoring), a GPS system (global positioning system) that alarms the principal company for any temperature variation during transportation, and a data logger system. In Pakistan, the VVM and GPS method is not implemented, while data logger use is also very limited.*”

(Regt 01)

“*In manufacturing industries, we have proper storage and temperature monitoring gadgets for vaccines such as data loggers that provide us with non-editable data. As you know that most of the vaccines are imported, at that level we have poor controls and policies regarding storage and transportation of vaccines from the importer to end-user; it’s very compromised.*”

(Mfr 06)

*“I think policies should be devised for vaccine temperature monitoring during storage and transportation. Currently, we are not well equipped for vaccines in terms of both legal policies and temperature control.*”

(Dist 10)

“*DRAP and drug inspectors should take appropriate measures in introducing proper temperature-controlled vehicles for the delivery of pharmaceutical products and vaccines.*”

(HP 09)

“*In my opinion, when we talk about chains of pharmacies with well-reputed names, although they tried to store vaccines at their required temperature, most of them are still lacking in utilizing data loggers for proper recording of temperature throughout vaccine storage.*”

(CP 05)

#### 3.6.3. Delayed Supply Chain Process

In view of most of the stakeholders, the shortage of qualified pharmacists, followed by inappropriate forecasting methods and funds, was the primary reason for the delayed supply chain process.

“*Lack of a qualified pharmacist in the supply chain management process is the major barrier toward a delayed supply chain process.*”

(Regt 05)

“*One of the major barriers to an effective supply chain process that delays supplies is poor planning and forecasting methods, either at the institution or at the manufacturing level.*”

(Mfr 05)

“*At times, there are issues at the manufacturer end as they are facing issues with the availability of raw material. Besides this, the dollar price fluctuates, which affects the price of pharmaceuticals, and, in this way, supplies are delayed.*”

(Dist 01)

“*Delays in the placement of orders, shortage of product raw materials, and fluctuation in dollar price are reasons responsible for effecting the supply chain process.*”

(HP 02)

“*The most common challenges towards effective supply chain management at the end of the supplier are inefficient transportation systems. They have specific days for booking and deliveries and don’t facilitate in case of any emergencies except those days.*”

(Pharm 01)

#### 3.6.4. Influence of Legal Policies

According to some regulators’ opinions, legal requirements positively influence the supply of pharmaceuticals. Legal policy for implementing an effective supply chain management system was not seen as a barrier by most pharmacists working in the manufacturing sector; however, some suggested that a few legal policies were creating issues for the supply chain management system of pharmaceutical products. Almost all of the distributors and community pharmacists highlighted that there are no legal policies that affect the implementation of effective supply chain management. Most hospital pharmacists thought that there is no influence of legal policy or requirement on the pharmaceutical supply chain process. In contrast, some pharmacists believed that legal policy influences the import of unregistered drugs and those drugs that are already registered in Pakistan but not available.

“*In my opinion, legal policies are for better control, and I don’t think that there is any legal policy that influences the consistency of supply of pharmaceuticals.*”

(Regt 04)

“*In my opinion, I don’t think any legal policy can affect the supply chain system. DRAP has taken major initiatives for improvements; import and export policies are especially very clear.*”

(Mfr 09)

“*I don’t think any legal policies influence the consistency of pharmaceutical supply chain at the distributor level.*”

(Dist 01)

“*I don’t think any legal policy influences the supplies of pharmaceutical products.*”

(HP 02)

“*Yes, I think there is little influence of legal policies or requirements on the consistency of supplies, especially for products that a hospital is directly importing for its patients. There are a number of DRAP and customs department requirements that take a lot of time to release that product, and, at the same time, we also face drug stock-outs.*”

(HP 01).

“*I don’t see any legal policy as a challenge for an effective supply chain process.*”

(CP 06).

#### 3.6.5. Lack of Integrated Supply Chain System

Most of the regulators considered the coordination among all stakeholders involved in the supply chain system as very poor. Another challenge highlighted by most of the pharmacists working in the manufacturing sector toward an effective supply chain was the lack of integration. Most of the distributors and community pharmacists stated that they have a good integrated supply chain and coordination system. Most hospital pharmacists highlighted that integration and coordination among supply chain systems are lacking and could be resolved by developing an efficient integrated system.

“*Good collaboration among all stakeholders is required for an efficient supply chain system but, unfortunately, we don’t have that effective collaboration.*”

(Regt 03)

“*Unfortunately, supply chain system is not efficient in Pakistan due to which we are facing problems.*”

(Mfr 07)

“*We have very good collaboration with the parent companies and institutes as well.*”

(Dist 05)

“*In my opinion, collaboration among all those involved in supply chain process is satisfactory, but not efficient.*”

(HP 04)

“*We have a good working relationship with our suppliers.*”

(CP 10)

#### 3.6.6. Strict Legal Requirement for Supply Chains

Most of the regulators were of the view that they did not face any challenges while complying with the legal requirements. However, some of them stated that they had issues in implementing strict legal requirements, and some pharmacists thought that a few legal policies were creating issues for the supply chain management system of pharmaceutical products. Although almost all of the distribution sector respondents were of the opinion that they had no issue in following the legal requirements, a few of them stated that legal requirements for a vaccine distributor must be different, and a normal distributor must not be allowed to deal with vaccine storage and delivery. Moreover, some hospital pharmacists were of the opinion that there should be no flexibility for compliance with legal requirements of pharmaceuticals, especially vaccines. Some pharmacists were of the view that legal requirements for local purchase of medicines with a defined limit of 2 km at Tehsil Headquarter Hospital (THQ) level are difficult for compliance. Most of the community pharmacists were of the opinion that they had legal policies, but unfortunately these are not implemented in Pakistan, which is again a challenge for the effective supply chain management.

“*No, I don’t think so we face any challenges in the implementation of legal requirements. The checklist that we follow for document evaluation or scrutiny (in DRAP) is in line with international practices, such that there should be no conflict of interest, as import and export are sensitive matters.*”

(Regt 04)

“*In my opinion, I don’t think any legal policy can affect the supply chain system. The DRAP has taken major initiatives for improvements; import and export policies are especially very clear.*”

(Mfr 09)

*“I don’t find any legal requirement as a problem for compliance.*”

(Dist 04)

“*We don’t face any challenges in implementing legal requirements. In my opinion, the legal requirements must be strictly implemented and monitored. There should be no flexibility for compliance with legal requirements as these are for life-saving products.*”

(HP 13)

“*Yes, we are facing problems in case of medicine and pharmaceutical purchases, as there is a legal limitation of 2 km radius at a THQ hospital level. We are legally bound to purchase medicines within the radius of 2 km from an A-category pharmacy, which we can hardly find within the defined geographical limits, and, if found, then they are not willing to supply us.*”

(HP 06)

“*As a qualified pharmacist, I personally like all the laws and believe that they must be implemented but, unfortunately, this is not the case at present.*”

(CP 09)

### 3.7. Theme 6: Strategies for Improving the Supply Chain in Pakistan

All the stakeholders suggested multiple strategies for improving supply chain management processes at the national level, including involvement of qualified pharmacists in supply chain departments, development of specific policies, the establishment of separate designated supply chain departments with separate budgets for improving the supply chain process, and improving coordination among all stakeholders.

“*In my view, DRAP should take a lead role for creating awareness regarding supply chain management and importance of pharmaceutical supply chain management in the healthcare system among all stakeholders, including provincial health departments, provincial governments, hospitals, and the pharmaceutical industry*.”

(Regt 01)

“*Cost-effective planning and forecasting are required by involving the relevant departments like marketing, procurement, and sales.*”

(Mfr 05)

“*At the manufacturing level, we should focus on producing specific products rather than getting registration of multiple products. Each pharmaceutical product is getting a share from the market in terms of number of packs sold. DRAP should also control this so that every pharmaceutical focused on developing market of specific products, as it will be easy to manage supply chain process efficiently.*”

(Mfr 03)

“*In my opinion, more qualified and competent people must be involved for better coordination and control of supply chain process.*”

(Dist 07)

“*I must say, at the hospital level, decision and policy making regarding inventory, purchase, or formulary should be performed by involving relevant qualified persons.*”

(Hosp 02)

“*There is a need to develop awareness among all healthcare professionals regarding the importance of pharmacists’ roles in hospital and pharmaceutical supply chain management.*”

(HP 04)

“*Proper temperature-controlled vehicles should be used. Cold supply chain, i.e., from manufacturer to distributor and then distributor to the pharmacies and then to end users must be properly maintained. Proper temperature maintenance must be ensured during the whole supply chain.*”

(CP 01)

## 4. Discussion

An efficient and consistent pharmaceutical supply chain process is responsible for delivering good-quality pharmaceutical products and services to the right consumer at the right time and place, which ultimately results in cost-efficient healthcare outcomes [6]. The present study reported that Pakistan’s supply chain system for pharmaceutical products and vaccines is currently not up to the mark. The issues are multifaceted, and the system is limited in terms of efficiency and cost-effectiveness. As an efficient pharmaceutical supply chain improves health outcomes, saves cost, and ensures timely availability of drugs [26], it raises intriguing questions regarding the need for policymakers in Pakistan to find suitable interventions to empower the healthcare system alignment with the local resources and challenges.

An operational framework of supply chain management, especially a pharmaceutical chain management system, is crucial throughout the complete supply chain cycle [27,28]. The present study results revealed that most of the respondents reported that SOPs, tools, and government guidelines are present. Generalized rules and regulations are also available and implemented as needed. This result may explain why pharmacists are less likely to be considered the most suitable profession in chain management by non-pharmacist logisticians [29]. Periodic revision, updating, and implementation of supply chain policies are very important due to advancement in the field of pharmaceuticals. The current study results reported that most of the respondents highlighted that, although policies are available, they require strict implementation and periodic revision. Most of the participants agreed that the pharmaceutical supply chain policies must be revised on an annual basis or as per requirements. Supplier selection and evaluation for timely delivery of pharmaceutical products and vaccines play a major role in the supply chain process. The present study results showed that a supplier is selected on the basis of their quality of services, timely delivery of supplies, market reputation, experience, financial conditions, authorization, and registration with a licensing authority as a supplier. Similar findings were reported from a study conducted in Bosnia and Herzegovina. The basic criteria used for supplier selection were the quality of services, timely deliveries, cost of the products, financial capacity, and professional dealing [30].

At the same time, supply chain management is a simple system used to maintain pharmaceutical drugs supply to hospitals and pharmacies [31]. In order to promote an efficient pharmaceutical supply chain process, key factors such as the procurement process, financial capabilities, stakeholder’s coordination, and training programs must be in place. The present study results reported that most of the respondents agreed that good inventory control, financial strengths, effective training programs, and coordination result in an efficient supply chain process, but training and skill development programs are missing at the level of regulators, some hospitals, and distributors. Although coordination among all stakeholders is implemented, the effectiveness is questionable and needs to be strengthened. The current study reported that most of the respondents believed that vaccine availability is one of the most significant and foremost issues in Pakistan that needs to be addressed. All of the respondents highlighted multiple factors responsible for a poor vaccine supply chain, including pricing issues, poor inventory management and forecasting, and raw material availability issues. Another qualitative study performed in Pakistan also reported similar results [32]. One of the key challenges in vaccine management is the maintenance and effective monitoring of the cold chain of vaccines from its manufacturing to storage and transportation to the end-users. Most of the respondents reported that cold chain maintenance of vaccines is a major issue, and that no appropriate temperature recording and monitoring system during the transportation is available. Moreover, vehicles utilized for the distribution of vaccines were also not up to the mark. Similar findings were reported in other countries and raised questions regarding the extent of safety and protection received by the patients [33,34,35,36], as well as the involvement of more professional and qualified personnel, periodic training (continuous professional development), development of regulations and policies for ensuring cold chain maintenance of vaccines, and promotion of effective coordination among all stakeholders.

Improved health outcomes and cost-saving are the result of an efficient supply chain process. The present study revealed that almost all stakeholders agreed that cost-effective health outcomes are achieved through a consistent and efficient supply chain management system. To improve the system, awareness and proper implementation of the pharmaceutical supply chain process are required. Similar findings were highlighted in a study conducted in the United Arab Emirates (UAE), which reported that improved supply chain management could enhance the efficacy in the healthcare sector and promote customer satisfaction [37]. Moreover, a systematic review of evidence from low-income and middle-income countries also confirmed the current study findings [26].

## 5. Conclusions

In Pakistan, all parts of the pharmaceutical supply chain have many unsolved problems, which reduce accessibility to medications and vaccines, eventually affecting public health. Among these parts, strict implementation of the laws, appropriate forecasting, inventory control, traceability of delayed order placement, training, involvement of professionally qualified staff, adequate financing and procurement process, and proper coordination and integration were noted by all stakeholders. Effective strategies need to be developed to make the pharmaceutical supply chain more resilient, adhering with the current regulations and demands. In this regard, policymakers need to deliberate on the integration of pharmaceutical supply chain activities with modern technologies, especially in the case of large-scale pharmaceutical industries, taking into account the business complexity, economic development, strong competition, rapid changes in customer needs, and a professional relationship among manufacturers, distributors, prescribers, and insurance organizations as buyers.

## 6. Recommendations

There is an urgent need to recruit more qualified staff at the level of regulation, manufacturing, distribution, hospitals, and community pharmacies for better control of pharmaceutical and vaccine supply chain processes. Periodic training and continuous skill development programs should be mandatory for all the staff involved in Pakistan’s pharmaceutical and vaccine supply chain. All stakeholders should participate in updating the policy for pharmaceutical supply chain management so that it may be practically implemented to support the access of medicines to the community.

## 7. Limitations

This study had several limitations that must be noted. First, it is better to triangulate the qualitative data obtained through interviews with other types of possible data, such as national documents and reports. Second, the views of the community as end-users may supplement the results, which should be considered in future studies. The findings of this study are not generalizable to large segments of the population, as we included only a small sample size of regulators, manufacturers, distributors, hospitals, and community pharmacists. Additionally, the participants were restricted to only two cities in Pakistan. Therefore, extrapolation of the results of this study beyond these two cities should only be performed with caution [38]. As with any studies that employ the qualitative interview method for data collection, the results from this study were further limited by the preconceived ideas of the interviewers toward the participants’ personal values. However, this interview bias was minimized as the participants’ anonymity was preserved using unique identification codes.

## 8. Significance of Our Study

This study identified some of the main factors which affect the supply chain management of pharmaceutical products and vaccines in Pakistan. Using the study findings, a well-organized master plan could be developed at the national level with the capability of integrating procurement, production, and distribution activities in Pakistan for achieving a competitive advantage in today’s marketplace. Low-income countries, especially those located in Southeast Asia, share a similar health infrastructure and challenges in the implementation of effective supply chain system for pharmaceutical products and vaccines. Therefore, these countries can also consider the findings of the current study as baseline data for revamping their supply chain models.

## Figures and Tables

**Table 1:** Perceptions of stakeholders regarding factors affecting supply chain management of pharmaceuticals products and vaccines in Pakistan.

Indicators	Regulators	Manufacturers	Distributors	Hospitals	Community Pharmacies
**Operational Framework of Supply Chain Management**					
Availability of government guidelines/policies	Available	Available	Not available	Partially available	Not available
Availability of SOPs	Available	Available	Available	Available	Not available
Availability of checklist for supply chain process	Available	Available	Available	Available	Available
**Key Indicators for Supply Chain Management**					
Selection criteria for supplier	Available	Available	Available	Available	Decided by pharmaceutical company
Availability of performance evaluation criteria	Available	Available	Available	Available	Available
**Factors Effecting Supply Chain Management in Pakistan**					
Procurement process	Strongly agreed	Strongly agreed	Strongly agreed	Strongly agreed	Strongly agreed
Financial resources	Strongly agreed	Strongly agreed	Strongly agreed	Agreed	Strongly agreed
Supplier institute relationship	Strongly agreed	Strongly agreed	Strongly agreed	Partially agreed	Strongly agreed
Consumer satisfaction	Strongly agreed	Strongly agreed	Strongly agreed	Strongly agreed	Strongly agreed
Trainings and skill development programs	Partially available	Available	Not available	Partially available	Not available
**Outcomes of Effective Supply Chain Management**					
Improve healthcare outcomes	Strongly agreed	Strongly agreed	Strongly agreed	Strongly agreed	Agreed
Reduce healthcare cost	Strongly agreed	Agreed	Strongly agreed	Strongly agreed	Agreed
**Current Challenges for Effective Supply Chain Management System in Pakistan**					
Issues related to import and availability of vaccine	Strongly agreed	Strongly agreed	Agreed	Strongly agreed	Agreed
Storage and temperature control of vaccine	Strongly agreed	Partially agreed	Agreed	Strongly agreed	Agreed
Delayed supply chain process	Lack of qualified staff and poor forecasting	Poor planning and forecasting	Raw material shortage and increase in dollar rate	Delayed order placement and political issue	Inefficient transportation and specific days for order booking
Influence of legal policies	Partially agreed	Partially agreed	Disagree	Partially agreed	Partially agreed
Coordination in supply chain system	Poor coordination	Poor coordination	Good coordination	Partial coordination	Partial coordination
Strict legal requirement for supply chain	No	Partial	No	Partial	No implementation
**Overall Perceptions of Stakeholders Regarding Supply Chain Management in Pakistan**					
	Unsatisfied	Satisfied	Satisfied	Partially satisfied	Partially satisfied

## Data Availability

All data generated during the study are presented in this paper and the Supplementary Materials.

## Supplementary Materials

The following supporting information can be downloaded at https://www.mdpi.com/article/10.3390/healthcare10091738/s1, Supplementary Table S1. Demographic characteristics of the participants.
