# Supplementary material for: Stakeholders’ Perspectives Regarding Supply Chain System of Pharmaceuticals and Vaccines in Pakistan: A Qualitative Study
Source: Healthcare (Basel). 2022 Sep 10;10(9):1738. doi: 10.3390/healthcare10091738 (PMC9498743; doi:10.3390/healthcare10091738)
Supplement: Supplementary file 1 [file healthcare-10-01738-s001.zip › healthcare-1848311-supplementary.pdf]

Supplementary Table S1. Demographic Characteristics of the Participants

Code	Gender	Qualification	Work Experience	Current Designation	Department	Number of Personnel
**Regulators**						
Regt 01	Male	B. Pharmacy & M.Phil.	16 years	Drug Inspector	District Health Office	4 persons
Regt 02	Male	B. Pharmacy & M.Phil.	15 years	Drug Controller	District Health Office	5-6 persons
Regt 03	Male	B. Pharmacy & M.Phil.	13 years	Deputy Drug Controller	District Health Office	5-6 persons
Regt 04	Male	Pharm-D & M.Phil.	04 years	Assistant Director	Drug Regulatory Authority of Pakistan	7-8 persons
Regt 05	Male	Pharm-D	03 years	Assistant Director	Drug Regulatory Authority of Pakistan	7-8 persons
Regt 06	Male	B. Pharmacy & M.Phil.	33 year	Head of Vaccine Production	National Institute of Health	3-4 persons
**Manufacturers**						
Mfr 01	Male	B. Pharmacy, M.Phil.	13 years	Production manager	Production	06 person
Mfr 02	Male	B. Pharmacy	15 years	Import and export manager	Import and export	05 person
Mfr 03	Male	B. Pharmacy, PHD	14 years	QC in charge	Quality Control	07 person
Mfr 04	Male	B. Pharmacy	17 years	GM production	Production	08 person
Mfr 05	Male	B. Pharmacy, M.Phil.	17 years	GM institutions	Regulatory	07 person
Mfr 06	Male	Pharm D	07 years	QC officer	Quality Assurance	06 person
Mfr 07	Male	B. Pharmacy, PHD	18 years	GM import and export	Import and Export	05 person
Mfr 08	Male	B. Pharmacy, M.Phil.	15 years	Head of QC	Quality Control	05 person
Mfr 09	Male	B. Pharmacy	14 years	Production manger	Production	06 person
**Distributors**						
Dist 01	Male	MBA	18 years	CEO	Supply chain	5
Dist 02	Male	Masters	16 years	Managing director	Supply chain	7
Dist 03	Male	MBA, MS, LLB	13 years	Institutional manager	Institutional department	13
Dist 04	Male	B. Pharmacy, M.Phil., PHD	16 years	General manager	Institutional department	6
Dist 05	Male	PharmD	9 years	Managing director	Retail department	7
Dist 06	Male	MBA	15 years	CEO	Institutional department	7
Dist 07	Male	Masters	12 years	Institutional manager	Retail department	9
Dist 08	Male	PharmD, M.Phil.	11 years	Institutional manager	Supply chain	15
Dist 09	Male	BSC	17 years	Marketing manager	Institutional department	6
Dist 10	Female	BSc	08 years	Supply chain manager	Retail department	6
Dist 11	Male	BSc	27 years	CEO	Institutional department	7
Dist 12	Male	PharmD, M.Phil.	07 years	General manager	Supply chain	7

Dist 13	Male	Masters	15 years	Marketing manager	Supply chain	9
**Hospital Pharmacy**						
Hosp 01	Female	Masters	10 years	Procurement officer	Procurement department	06
Hosp 02	Female	PharmD	5 years	Hospital Pharmacist	Store Department	08
Hosp 03	Male	PharmD.	08 years	Hospital Pharmacist	Pharmacy	05
Hosp 04	Male	PharmD, M.Phil.	09 years	Hospital Pharmacist	Store department	07
Hosp 05	Male	PharmD.	06 years	Hospital Pharmacist	Pharmacy	07
Hosp 06	Male	PharmD, M.Phil.	02 years	Procurement Officer	Procurement department	04
Hosp 07	Female	B. Pharmacy, M.Phil. PHD	14 years	Senior Hospital Pharmacist	Pharmacy	10
Hosp 08	Male	PharmD, M.P.H	11 years	Procurement Manager	Pharmacy	15
Hosp 09	Male	PharmD	04 Years	Hospital Pharmacist	Pharmacy	07
Hosp 10	Male	PharmD	07 years	Hospital Pharmacist	Pharmacy	11
Hosp 11	Male	PharmD, M.Phil.	11 years	Hospital Pharmacist	Pharmacy	07
Hosp 12	Female	PharmD	09 years	Senior Pharmacist	Pharmacy	10
Hosp 13	Male	PharmD, M.Phil.	05 years	Hospital pharmacist	Pharmacy	09
**Community Pharmacy**						
Pharm 01	Female	PharmD	04 years	Pharmacist	Pharmacy	08
Pharm 02	Male	PharmD	07 years	Senior pharmacist	Pharmacy	07
Pharm 03	Male	Pharm D	03 years	Pharmacist	Pharmacy	07
Pharm 04	Male	Pharm D M.Phil.	06 years	Manager pharmacy	Pharmacy	05
Pharm 05	Male	Pharm D	05 years	CEO pharmacy	Pharmacy	04
Pharm 06	Male	Pharm D	03 years	In charge pharmacy	Pharmacy	06
Pharm 07	Male	Pharm D M.Phil.	02 years	Pharmacist	Pharmacy	05
Pharm 08	Male	Pharm D	05 years	Senior pharmacist	Pharmacy	05
Pharm 09	Male	Pharm D M.Phil.	04 years	Manager Pharmacy	Pharmacy	05
Pharm 10	Male	Pharm D	02 years	Pharmacist	Pharmacy	04
Pharm 11	Male	Pharm D	06 years	In charge Pharmacy	Pharmacy	04
Pharm 12	Male	Pharm D	04 years	Pharmacist	Pharmacy	06
Pharm 13	Male	Pharm D	05 years	Pharmacist	Pharmacy	05
Pharm 14	Male	Pharm D	04 years	Pharmacist	Pharmacy	05
Pharm 15	Male	B. Pharmacy PHD	15 years	CEO pharmacy	Pharmacy	06

Regt: Regulator, Mfr: Manufacturers, Dist: Distributor, HP: Hospital pharmacist, CP: Community pharmacist
